# Higher blood manganese level associated with increased risk of adult latent tuberculosis infection in the US population

**DOI:** 10.3389/fpubh.2024.1440287

**Published:** 2024-07-24

**Authors:** Min Qi, Huan Zhang, Jian-Qing He

**Affiliations:** ^1^Department of Pulmonary and Critical Care Medicine, West China Hospital, Sichuan University, Chengdu, China; ^2^State Key Laboratory of Respiratory Health and Multimorbidity, West China Hospital, Sichuan University, Chengdu, China

**Keywords:** National Health and Nutrition Examination Survey, heavy metals, manganese, latent tuberculosis infection, adult

## Abstract

**Background:**

The associations between blood heavy metal levels and latent tuberculosis infection (LTBI) have not been fully elucidated. The aim of this study was to investigate the potential association between blood heavy metal levels and LTBI in adults using National Health and Nutrition Examination Survey data from 2011 to 2012.

**Methods:**

We enrolled 1710 participants in this study, and compared the baseline characteristics of participants involved. Multivariate logistic regression analysis, restricted cubic splines (RCS) analysis, along with subgroup analysis and interaction tests were utilized to explore the association between blood manganese (Mn) level and LTBI risk.

**Results:**

Participants with LTBI had higher blood Mn level compared to non-LTBI individuals (*p* < 0.05), while the levels of lead, cadmium, total mercury, selenium, copper, and zinc did not differ significantly between the two groups (*p* > 0.05). In the fully adjusted model, a slight increase in LTBI risk was observed with each 1-unit increase in blood Mn level (OR = 1.00, 95% CI: 1.00–1.01, *p* = 0.02). Participants in the highest quartile of blood Mn level had a threefold increase in LTBI risk compared to those in the lowest quartile (OR = 4.01, 95% CI: 1.22–11.33, *p* = 0.02). RCS analysis did not show a non-linear relationship between blood Mn level and LTBI (non-linear *p*-value = 0.0826). Subgroup analyses and interaction tests indicated that age, alcohol consumption, and income-to-poverty ratio significantly influenced LTBI risk (interaction *p*-values<0.05).

**Conclusion:**

Individuals with LTBI had higher blood Mn level compared to non-LTBI individuals, and higher blood Mn level associated with increased LTBI risk.

## Introduction

Tuberculosis (TB), caused by the bacillus *Mycobacterium tuberculosis*, stands as one of the leading causes of global mortality (1). Recently, studies have definitively shown that a continuous spectrum of bacterial metabolic activity and opposing immunological responses transpire within human TB infection, spanning from latent infection to active disease (2, 3). It has been documented that roughly a quarter of the global population is estimated to have been infected with TB, manifesting as latent TB infection (LTBI) (4). Acting as the most extensive reservoir for active TB, only a small proportion of LTBI individuals, around 5–10%, will progress into active TB over their lifetimes (5), and the precise mechanisms behind this transformation remain inadequately understood. If left unaddressed, the current LTBI burden alone could potentially hinder the attainment of global TB elimination targets (4). The initial interactions between mycobacterial and the host’s innate immune system substantially dictate the establishment of TB infection and the progression of disease development (6).

Heavy metals are characterized by their high density and potential toxicity to living organisms. They can be found naturally in the environment, but human activities can lead to elevated levels of heavy metals in the body, such as heavy metals in the dust can be inhaled and caused ecological risk (7). Notably, certain heavy metals such as manganese (Mn), lead (Pb), cadmium (Cd), mercury (Hg), and selenium (Se) have attracted attention in correlation with diseases due to their potential to modulate the immune response (8). A study showed that exposure to heavy metals like Pd and Hg may cause severe diseases including but not limited to urinary tract cancer (9). Juttukonda et al. (10) demonstrated that elevated dietary Mn levels intensified *S. aureus* virulence and infection of the murine heart. Moreover, Seth et al. (11) discovered that a single oral dose of Cd or Mn heightened the susceptibility of mice to a sub-lethal viral infections, resulting in increased symptom severity and mortality. These heavy metals have been shown to impact immune functions (12), oxidative stress levels (13), and inflammatory responses (14), all of which are factors relevant to tuberculosis infection. Mn is an essential trace element and involved in many metabolic processes of nutrients and life activity (15). The levels of manganese are either too low or too high can cause adverse health effects (15). *In vitro* experiment and bioinformation analysis found that Mn^2+^ could enhance the necrosis of macrophage and suppress the survival of Mycobacterial strains via activation of tumor necrosis factor signal pathway (16, 17). A survey in South Africa revealed that workers in manganese mines with the highest prevalence of TB and other diseases (18). However, the association between heavy metal levels, especially Mn, on TB infection has not been previously elucidated.

The purpose of this study is to compare the levels of blood heavy metal in different TB infection status and explore the potential connection between blood Mn and latent tuberculosis infection in adults within the United States population, utilizing data sourced from the National Health and Nutrition Examination Survey (NHANES) conducted during 2011–2012.

## Materials and methods

### Study population

The analytic sample was obtained from the NHANES, a national cross-sectional study centered on the U.S. population, aiming at evaluating the health and nutritional status of Americans. Participants underwent standardized interviews at their residences and health assessments at mobile examination centers to evaluate their physical status and laboratory-related data. The research protocol for NHANES received approval from the National Center for Health Statistics (NCHS) Research Ethics Review Board. All participants provided written informed consent. Comprehensive information on the NHANES study design and data can be accessed publicly at https://www.cdc.gov/nchs/nhanes/.

**Figure 1:**
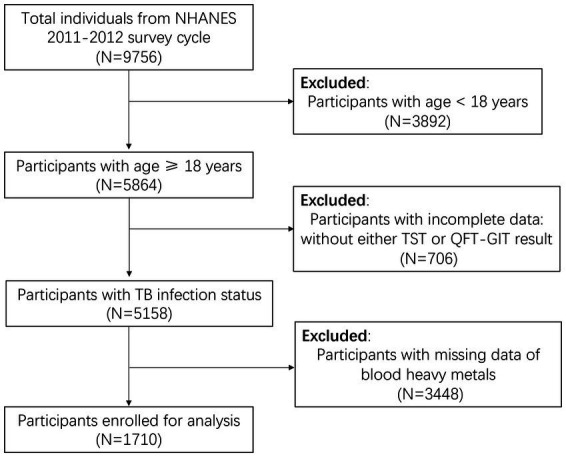
Flowchart of the individuals enrolled from National Health and Nutrition Examination Survey (NHANES) 2011–2012. LTBI, latent tuberculosis infection; TB, tuberculosis; QFT-GIT, QuantiFERON-TB Gold in Tube test; TST, Tuberculin skin test.

We employed the 2011–2012 NHANES survey cycle to assess the levels of heavy metals in different LTBI statuses and explore the association between blood Mn level and TB infection among adults, as this cycle exclusively contained complete latent TB screening data. LTBI data was based on tuberculin-skin-test (TST) and interferon-gamma release assay (IGRA), with participants under 18 years of age being excluded. Our study initially encompassed 9,756 participants, subsequent excluded those under 18 years (*n* = 3,892), those lacking TST or QuantiFERON-TB Gold in Tube test (GFT-GIT) results (*n* = 706), and those without data on blood heavy metals levels (*n* = 3,448). Ultimately, 1710 individuals enrolled in our analysis (Figure 1).

### Assessment of blood heavy metals level

Venous blood samples were collected for the quantification of concentrations of Pb, Cd, total mercury (THg), Mn, Se in the form of whole blood specimens, copper (Cu) and zinc (Zn) of serum specimens in the mobile examination center in 30 different study locations of the United States from 2011 to 2012. These measurements were carried out using inductively coupled plasma mass spectrometry for whole blood specimens and inductively coupled plasma dynamic reaction cell mass spectrometry for serum specimens. The detection limit for all analytes remained consistent within the dataset. Pb has a lower detection limit of 0.25 μg/dL, Cd was 0.16 μg/L, Se was 30 μg/L, Mn was 1.06 μg/L, THg was 0.16 μg/L, Cu was 2.5 μg/dL and Zn was 2.9 μg/dL. In instances where the recorded result fell below the detection limit, the value assigned to that specific variable equated to the detection limit divided by the square root of two.

### The definition of LTBI

In our study, individuals with positive GFT-GIT results or positive TST were considered as having LTBI, and TST positive defined as an induration size of ≥ 10 mm for the TST, a commonly applied criterion for adults in the US, excluding individuals with special risk factors (19). To interpret a positive QFT-GIT outcome, we adhered to NHANES guidelines which entailed the following criteria: (1) The Nil value needed to be ≤8.0 IU gamma interferon (IF)/ml, (2) The TB antigen value minus the Nil value had to be ≥0.35 IU IF/ml, (3) The TB antigen value minus the Nil value had to be ≥25% of the Nil value. Furthermore, a subdued response to mitogen (< 0.5 IU/mL) was considered as suggestive of an indeterminate result when a blood sample also demonstrated a negative response to the TB antigens.

### Other variables

Our analysis also considered a range of variables, including but not limited to age, gender, race, body mass index (BMI), education level, marital status, alcohol consumption, smoking habits, the history of asthma, chronic obstructive pulmonary diseases (COPD), diabetes mellitus (DM), hyperlipidemia, hypertension, the income-to-poverty ratio (PIR), prior active tuberculosis, and TB medications. Moreover, we integrated laboratory measurements like albumin levels into analysis. DM was defined using a variety of criteria, including self-reported DM diagnosis, HbA1c levels ≥6.5%, fasting glucose levels ≥7.0 mmol/L, random blood glucose levels ≥11.1 mmol/L, two-hour plasma glucose levels ≥11.1 mmol/L in an oral glucose tolerance test, or the use of oral hypoglycemic agents or insulin. BMI was calculated by dividing weight in kilograms by the square of height in meters, rounded to one decimal place. Following the guidelines established by the World Health Organization, the BMI classifications were designated as: underweight <18.5 kg/m^2^, normal weight 18.5–24.9 kg/m^2^, overweight 25–29.9 kg/m^2^, and obesity ≥30 kg/m^2^. Comprehensive information concerning these variables can be accessed on the website: https://www.cdc.gov/nchs/nhanes/.

### Statistics

The statistical analysis adhered to NHANES analysis guidelines and included intricate sampling weights to account for multi-stage clustering surveys. Continuous variables were represented by mean values and standard deviations, while categorical variables were expressed as counts and proportions. The comparison of continuous variables employed the weighted Student’s *t*-test, while the comparison of categorical variables used the weighted chi-square test.

Due to a significant difference in blood Mn levels between the Non-LTBI and LTBI groups, we proceeded to assess the correlation between blood Mn levels (categorized into four levels) and LTBI using a multivariable regression model to adjust potential confounding variables. The Crude Model had no adjusted covariates. In Model 1, adjustments included age, gender, race, and education levels. Model 2 further adjusted for age, gender, race, education levels, alcohol consumption, and PIR. Additionally, an interaction test was utilized to analyze heterogeneity among subgroups.

To explore potential non-linear relationships within the regression models comprehensively, the Restricted Cubic Spline (RCS) analysis, was employed to assess the intricate associations between blood Mn level and the LTBI risk, incorporating four distinct piecewise points.

All analyses were performed using R version 4.2.2 (https://www.r-project.org/, The R Foundation). Statistical significance was determined by a threshold of *p* < 0.05.

## Results

### Baseline characteristics of participants

The weighted baseline characteristics of the individuals included were shown in Table 1. Our study encompassed 1710 participants, and their average age was 45.74 ± 0.92 years, of which 49.59% were female and 50.41% were male.

**Table 1:** The baseline characteristics and blood heavy metal levels in different TB statuses.

Variables	Total (*n* = 1710)	Non-LTBI (*n* = 1,478)	LTBI (*n* = 232)	*p-*value
Age	45.74 (0.92)	45.40 (1.00)	49.66 (1.37)	0.02
PIR	2.87 (0.11)	2.92 (0.11)	2.31 (0.18)	0.003
BMI group				0.88
Normal weight	529 (30.94)	455 (30.67)	74 (31.66)	
Obese	591 (34.56)	522 (33.40)	69 (32.07)	
Overweight	547 (31.99)	466 (33.37)	81 (32.71)	
Underweight	43 (2.51)	35 (2.56)	8 (3.56)	
Sex				0.004
Female	848 (49.59)	754 (52.72)	94 (39.20)	
Male	862 (50.41)	724 (47.28)	138 (60.80)	
Race				<0.0001
Mexican American	162 (9.47)	126 (6.19)	36 (17.78)	
Non-Hispanic Black	438 (25.61)	381 (10.57)	57 (14.92)	
Non-Hispanic White	623 (36.43)	596 (70.24)	27 (32.53)	
Others	487 (28.48)	375 (13.00)	112 (34.77)	
Marital				0.38
Divorced	156 (9.63)	132 (10.02)	24 (10.75)	
Living with partner	127 (7.84)	112 (8.27)	15 (6.19)	
Married	798 (49.26)	670 (52.94)	128 (55.96)	
Never married	364 (22.47)	334 (22.09)	30 (16.84)	
Separated	46 (2.84)	40 (1.74)	6 (2.36)	
Widowed	129 (7.96)	106 (4.94)	23 (7.91)	
Education level				0.01
College graduate or above	434 (25.39)	384 (32.83)	50 (27.71)	
High school graduate or less	782 (45.76)	655 (36.06)	127 (52.76)	
Some college or AA degree	493 (28.85)	438 (31.12)	55 (19.53)	
Alcohol Status				0.02
Former	256 (16.7)	207 (13.62)	49 (23.41)	
No	244 (15.92)	208 (10.56)	36 (12.23)	
Yes	1,033 (67.38)	914 (75.82)	119 (64.36)	
Smoking status				0.72
Former	370 (22.84)	310 (22.98)	60 (24.99)	
Never	940 (58.02)	820 (57.79)	120 (52.57)	
Now	310 (19.14)	264 (19.23)	46 (22.44)	
DM				0.22
DM	318 (18.79)	258 (13.77)	60 (19.58)	
IFG	45 (2.66)	36 (3.22)	9 (1.94)	
IGT	64 (3.78)	53 (4.34)	11 (6.16)	
No	1,265 (74.76)	1,114 (78.68)	151 (72.31)	
Asthma				0.54
No	1,462 (85.5)	1,262 (84.79)	200 (86.84)	
Yes	248 (14.5)	216 (15.21)	32 (13.16)	
COPD				0.61
No	1,544 (94.96)	1,326 (94.01)	218 (95.26)	
Yes	82 (5.04)	74 (5.99)	8 (4.74)	
Hyperlipidemia				0.75
No	557 (32.57)	496 (32.25)	61 (31.07)	
Yes	1,153 (67.43)	982 (67.75)	171 (68.93)	
Hypertension				0.39
No	1,040 (60.82)	909 (65.18)	131 (61.20)	
Yes	670 (39.18)	569 (34.82)	101 (38.80)	
Prescribed medicine for active TB				0.14
No	4 (25)	1 (58.99)	3 (28.26)	
Yes	12 (75)	4 (41.01)	8 (71.74)	
Lived in household TB sick person				<0.0001
No	1,638 (96.41)	1,428 (98.33)	210 (93.70)	
Yes	61 (3.59)	42 (1.67)	19 (6.30)	
Prescribed medicine for preventing TB				0.56
No	66 (54.55)	32 (60.47)	34 (53.56)	
Yes	55 (45.45)	26 (39.53)	29 (46.44)	
Ever told you had active TB				< 0.0001
No	1,683 (99.06)	1,465 (99.72)	218 (95.39)	
Yes	16 (0.94)	5 (0.28)	11 (4.61)	
Blood heavy metal concentrations				
Blood Pb (μmol/L)	0.07 (0.00)	0.07 (0.00)	0.07 (0.00)	0.3
Blood Cd (nmol/L)	4.29 (0.11)	4.24 (0.13)	4.83 (0.50)	0.31
Blood THg (μmol/L)	7.74 (0.90)	7.46 (0.97)	11.07 (1.99)	0.13
Blood Se (μmol/L)	2.47 (0.02)	2.47 (0.02)	2.45 (0.03)	0.48
Blood Mn (μmol/L)	175.93 (2.22)	174.51 (2.16)	192.38 (6.13)	0.01
Serum Cu (μmol/L)	18.44 (0.22)	18.46 (0.23)	18.17 (0.37)	0.48
Serum Zn (μmol/L)	12.74 (0.10)	12.78 (0.11)	12.26 (0.23)	0.06

Continuous variables were summarized using mean (SE), while categorical variables were presented as counts (percentages). LTBI, latent tuberculosis infection; PIR, income-to-poverty ratio; BMI, body mass index; DM, diabetes mellitus; IFG, impaired fasting glycemia; IGT, impaired glucose tolerance; COPD, chronic obstructive pulmonary disease; TB, tuberculosis; Mn, manganese; Pb, lead; Cd, cadmium; THg, total mercury; Se, selenium; Cu: copper; Zn: zinc.

Compared with non-LTBI participants, the LTBI group exhibited older age, a higher proportion of males, lower PIR, an increased representation of other racial backgrounds, lower educational attainment, greater alcohol consumption, and a heightened prevalence of previous contact with a household TB member and previous active TB history (all *p* < 0.05). Conversely, no statistically significant differences emerged in terms of marital status, smoking habits, DM, asthma, COPD, prior TB medication, hyperlipidemia, hypertension and BMI categories (all *p* > 0.05).

In regard to blood heavy metal levels, a significant discrepancy was observed in LTBI individuals, who demonstrated higher levels of Mn compared to their non-LTBI counterparts (*p* < 0.05). However, levels of Pb, Cd, THg, Se, Cu, and Zn did not exhibit noteworthy variations between the two groups (*p* > 0.05).

### Higher blood Mn level increases the risk of LTBI

We also explored how blood Mn level related to the risk of LTBI using 3 different models (refer to Table 2). We identified a positive association between blood Mn level and LTBI risk across the crude, minimally adjusted, and fully adjusted models (*p* < 0.05). In the fully adjusted model, a marginal rise in LTBI risk was apparent with each 1-unit elevation in blood Mn level (OR = 1.00,95% CI: 1.00–1.01, *p* = 0.02). Then we further adjusted blood Mn level from a continuous variable to a categorical variable, and found that participants in the highest quartile of blood Mn level demonstrated nearly a threefold increase in LTBI risk compared to those in the lowest quartile (OR = 4.01, 95% CI: 1.22–11.33, *p* = 0.02).

**Table 2:** Multivariate logistic regression models of blood Mn level with LTBI risk.

	Blood Mn levels		
LTBI	Crude model	Model 1	Model 2
	OR (95% CI)	OR (95% CI)	OR (95% CI)
	*p*-value	*p*-value	*p*-value
Continuous	1.00 (1.00–1.01), *p* = 0.001	1.00 (1.00–1.00), *p* = 0.01	1.00 (1.00–1.01), *p* = 0.02
Categories			
Quartile 1	Reference	Reference	Reference
Quartile 2	2.80 (1.45,5.41), 0.005	3.05 (1.27,7.36), 0.02	3.15 (0.96,10.32), 0.06
Quartile 3	2.22 (1.28,3.84), 0.01	2.47 (1.19,5.11), 0.02	2.64 (1.01, 6.93), 0.05
Quartile 4	3.67 (2.12,6.37), <0.001	3.79 (1.76,8.17), 0.005	4.01 (1.42,11.33), 0.02

Crude model: no covariates were adjusted. Model 1: adjusted age, gender, race and education status. Model 2: adjusted age, gender, race, education levels, alcohol consumption, and PIR. LTBI, latent tuberculosis infection; PIR, income-to-poverty ratio; Mn, manganese; OR: odd ratio; CI: confidence interval.

**Figure 2:**
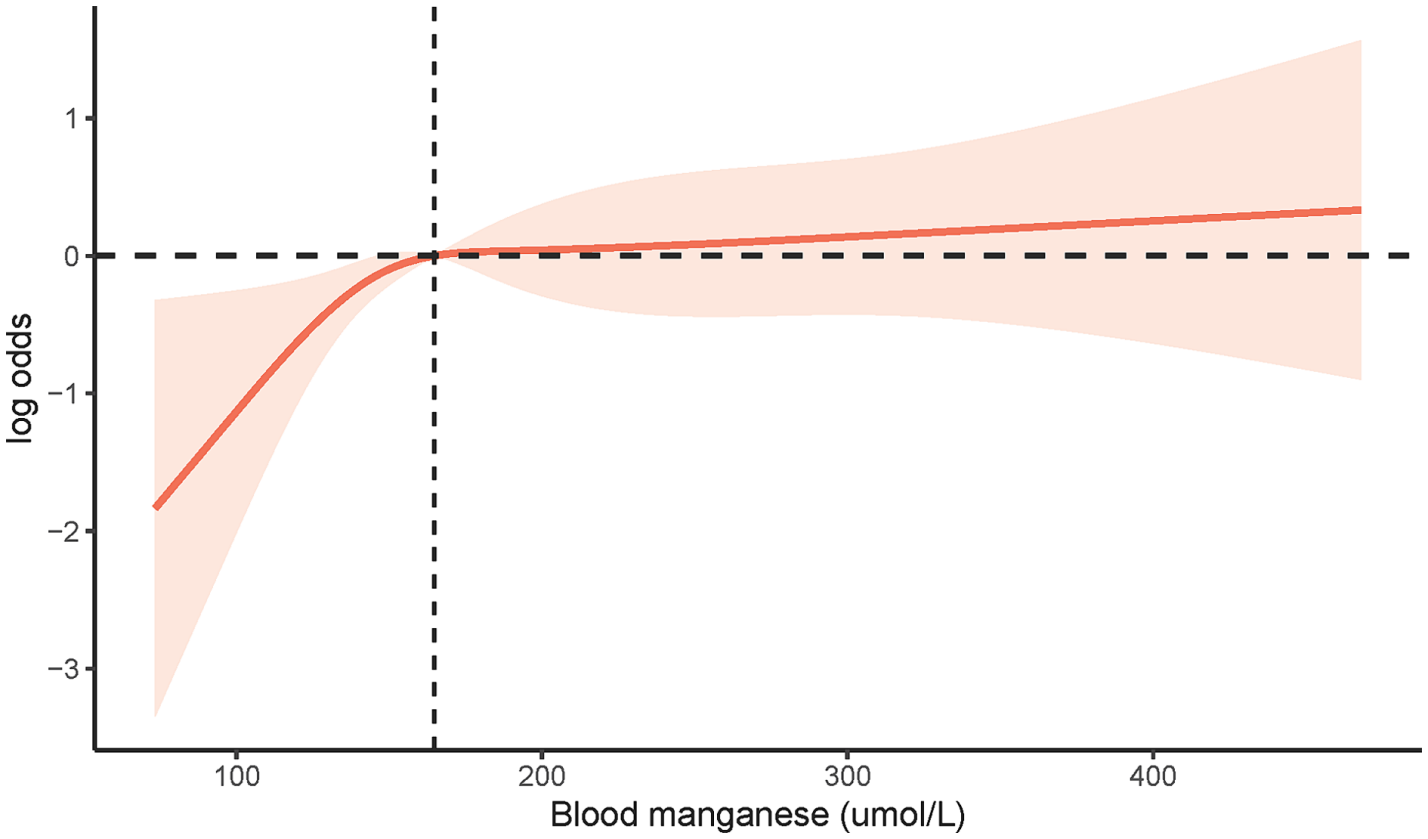
The restricted cubic spline analysis between blood Mn level and risk of LTBI (*p* non-linear = 0.0826). LTBI, latent tuberculosis infection; Mn, manganese.

To explore deeper into the correlation between blood Mn level and LTBI risk, we utilized Restricted Cubic Spline (RCS) analysis (refer to Figure 2). Our findings revealed no evidence of a non-linear correlation between blood Mn levels and LTBI (non-linear *p*-value = 0.0826).

### Subgroup analysis

**Figure 3:**
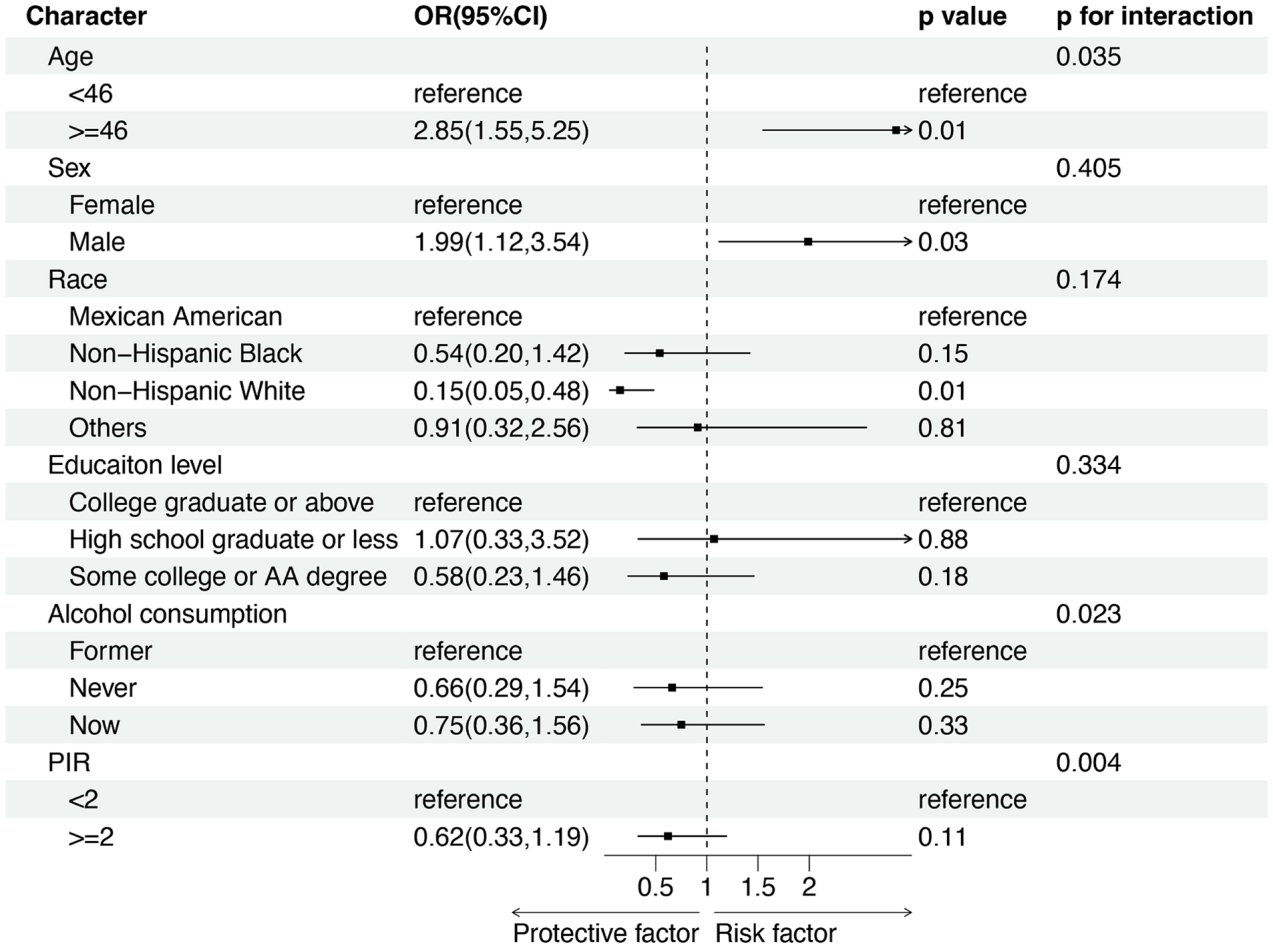
Subgroup analysis for the association between blood Mn level and risk of LTBI. PIR, income-to-poverty ratio. OR: odd ratio; CI: confidence interval.

In the subgroup analysis, we adjusted the potential continuous covariates into categorical variables based on their mean values (as depicted in Figure 3). Our findings indicated that individuals aged over 46-year-old and male exhibited an elevated risk of LTBI (OR = 2.85, 95% CI 1.55–5.25, *p* = 0.01; OR = 1.99, 95% CI 1.12–3.54, *p* = 0.03; respectively). Conversely, being of Non-Hispanic White ethnicity appeared to show protection against LTBI (OR = 0.15, 95% CI 0.05–0.48, *p* = 0.01). Furthermore, we explored the interaction between age, alcohol consumption, and PIR on the relationship between blood Mn level and LTBI risk, as indicated by interaction test on the association between blood Mn level and LTBI risk (*p* for interaction were 0.035, 0.023, and 0.004, respectively). Nevertheless, our interaction analysis revealed that gender, race, and educational level did not significantly modify the positive association between blood Mn level and LTBI risk (*p* for interaction >0.05).

## Discussion

In this cross-sectional study of 1710 participants, we discerned that individual with LTBI exhibited a notably elevated concentration of blood Mn compared to their non-LTBI counterparts. Conversely, blood levels of Pb, Cd, THg, Se, Cu, and Zn demonstrated no statistically significant difference between the two groups. We further analyzed the association between blood Mn level and LTBI risk, and found a positive association where heightened blood Mn levels were linked to an increased susceptibility to LTBI. Subsequent subgroup analyses and interaction assessments underscored the pivotal influence of age on the association between blood Mn level and LTBI risk.

To our knowledge, this is the inaugural investigation to assess the levels of blood heavy metals in different TB infection statuses and found the positive association between blood Mn level and LTBI risk. Mn^2+^ plays a critical role in cartilage and bone health, wound healing, mitochondria maintenance, glucose production, and urea cycle function of human health (20). In addition, Mn performs a vital role in host-pathogen interactions. Host can restrict invading microbes from utilizing Mn to combat infection (21), and Mn could enhance the releasing of cytokines of macrophage and serve as damage-associated molecular pattern that inhibited the proliferation of pathogens (16, 22). However, excessive Mn exposure, particularly in occupational settings, such as mining and welding, is known to induce adverse health effects, primarily affecting the central nervous system and lung tissue through inhalation exposure (23). A study in Korean revealed that compared with health control, active TB patients with elevated blood Mn level, but the difference was not statistically significant (24). Previous investigation of the mineworkers revealed that those wok in Mn mine with increased TB risk (18), however, the research did not detect the blood Mn level and the outcome may confounded by covariates socio-demographic factors like age, length of service and so on. In our study, after adjusting covariates, we found blood Mn level was associated with increased LTBI risk. Mn ions homeostasis is pivotal for host immune responses and innate immune activation, contributing substantially to antiviral defense (25). Studies have indicated that excessive Mn exposure in adults leads to a significant reduction in C3 levels (25). Furthermore, the study about the effects of welding and exposure to Mn on the cellular and humoral immune system has revealed that higher Mn exposure was associated with decreased CD19^+^ B lymphocytes (26), T lymphocytes, and specific T-lymphocyte sub-populations like CD8+ T cells (27). Given the crucial roles of innate and adaptive immunity in TB defense (28), particularly CD4+ T cells and CD8+ T cells (29), there is substantial evidence supporting the notion that CD4+ T cells release cytokines, such as interferon-γ (IFN-γ) in response to specific antigens of mycobacterial to enhance phagocytosis of macrophage to control TB (30). Moreover, recent study has also implicated TB-specific antibodies in TB exposure responses (31). Impaired cell-mediated and humoral immunity can heighten susceptibility to TB and compromise its control. Our investigation further revealed that age, alcohol consumption, and PIR interacted with the relationship between blood Mn levels and LTBI. Advanced age, alcohol consumption, and poverty were associated with weaken immune function and reduced ability to respond effectively to infections (32, 33). These factors may synergistically exacerbate the adverse effects of Mn exposure, further compromising individual immunity. Among the older adult, Mn level tend to accumulate with age and occupational exposure, resulting in weakened immune systems and heightened susceptibility to infections like TB.

Furthermore, our study disclosed that the levels of other heavy metals, such as Pb, Cd, Thg, Se, Cu, and Zn did not exhibit significant variations between LTBI individuals and their non-LTBI counterparts. Notably, Occupational exposure remains the primary cause of elevated Cd levels. Cd intake in humans occurs either through ingestion or inhalation, with cigarette smoking being a major source of exposure, increasing Cd levels in smokers up to four to five times those of non-smokers (34). Cd is known to cause cellular damage, impacting DNA repair, cellular enzyme activity and membrane structure (35), ultimately leading to lung emphysema and various tobacco-related lung diseases (36), However, our study did not reveal significant differences in tobacco exposure between the two groups, which may account for the lack of disparity in Cd levels. Similarly, elevated Pb levels are predominantly associated with occupational exposure. With higher Pb exposure potentially inducing an array of systematic deleterious effects, including hypertension, anemia, cognitive impairment, infertility, immune imbalances, development delays, vitamin D deficiency, and gastrointestinal issues (37). The modulation of immunity by Pb remains a subject of debate, with *in vitro* study indicating cytotoxic effects on immune cells and diminished cytokine production (38). While study in children suggested that Pb exposure increases the production of central memory T cells (39). Conversely, Hg bioaccumulates mainly through food consumption, particularly in fish containing methyl mercury (Me-Hg). Hg, a naturally occurring metal ubiquitous in the environment can lead to central nervous system injuries, renal dysfunction, gastroenterology ulceration, hepatotoxicity (14), and enhanced pro-inflammatory cytokine release in LPS-stimulated human peripheral blood mononuclear cells, thereby affecting the human immune system (40). However, in our study, there was no significant difference in blood Thg levels between LTBI and health control groups, which may be attributed to our measurement of Thg rather than Me-Hg and inorganic Hg, as these forms of Hg may exert different effects on immune function (40). Se is an essential component of antioxidant enzymes such as glutathione peroxidase and thioredoxin reductase, as well as thyroid hormone-converting deiodinase enzymes. Trace amounts of Se are vital for cellular function in all animals and are primarily obtained through dietary intake (41). Se plays a pivotal role in facilitating an effective immune response, controlling systemic inflammation, and maintaining overall human health (42). However, in the context of sepsis, no significant variation in blood Se levels was found between the sepsis and control groups (43), mirroring the results observed in our investigation. In summary, the similarity in blood levels of heavy metals such as Pb, Cd, Hg, and Se between individuals with LTBI and healthy individuals can be attributed to various factors, including environmental exposure, individual variability in metabolism and elimination, sample size limitations, confounding variables, and the timing of exposure. Further research involving larger, more rigorously controlled studies may be necessary to gain a more comprehensive understanding of potential associations between heavy metal exposure and LTBI.

Our study boasts several notable strengths. Firstly, it relies on data from NHANES, a nationally representative population-based sample, and the selection of samples and sample size are adequately representative. Secondly, we meticulously adjusted for confounding covariates to mitigate potential confounding bias, enhancing the reliability of our findings. Nevertheless, our study carries certain inherent limitations. First and foremost, the cross-sectional study design precludes us from establishing causality between blood heavy metal and LTBI risk. Secondly, excluding individuals without heavy metal concentration measurements would lead unavoidable selection bias. Thirdly, despite our adjustments for several potential covariates, we cannot completely rule out the possibility of unaccounted confounders, such as the use of specific medications and the presence of comorbidities. Finally, it is worth noting that the NHANES database exclusively comprises data from the US population, thus limiting the generalizability of our results to a broader international context.

## Conclusion

Individuals with LTBI had higher blood Mn level compared to non-LTBI individuals, while blood levels of Pb, Cd, THg, Se, Cu, and Zn did not differ significantly. Blood Mn level was linked to an increased susceptibility to LTBI risk, which were dependent on individuals’ age, alcohol consumption and PIR.

## Data availability statement

The datasets presented in this study can be found in online repositories. The names of the repository/repositories and accession number(s) can be found in the article/supplementary material.

## Ethics statement

The studies involving humans were approved by NCHS Research Ethics Review Board. The studies were conducted in accordance with the local legislation and institutional requirements. The participants provided their written informed consent to participate in this study.

## Author contributions

MQ: Conceptualization, Formal analysis, Project administration, Software, Visualization, Writing – review & editing. HZ: Conceptualization, Formal analysis, Investigation, Methodology, Writing – original draft. J-QH: Conceptualization, Project administration, Supervision, Validation, Writing – review & editing.

## References

[1] World Health Organization. Global tuberculosis report 2023. Geneva: World Health Organization (2023).

[2] Kendall EA Shrestha S Dowdy DW. The epidemiological importance of subclinical tuberculosis. A critical reappraisal. Am J Respir Crit Care Med. (2021) 203:168–74. doi: 10.1164/rccm.202006-2394PP, PMID: 33197210 PMC7874405

[3] Richards AS Sossen B Emery JC Horton KC Heinsohn T Frascella B. Quantifying progression and regression across the spectrum of pulmonary tuberculosis: a data synthesis study. Lancet Glob Health. (2023) 11:e684–92. doi: 10.1016/S2214-109X(23)00082-7, PMID: 36966785 PMC10126316

[4] Houben RM Dodd PJ. The global burden of latent tuberculosis infection: a re-estimation using mathematical modelling. PLoS Med. (2016) 13:e1002152. doi: 10.1371/journal.pmed.1002152 27780211 PMC5079585

[5] Yang Q Qi F Ye T Li J Xu G He X. The interaction of macrophages and CD8 T cells in bronchoalveolar lavage fluid is associated with latent tuberculosis infection. Emerg Microbes Infect. (2023) 12:2239940. doi: 10.1080/22221751.2023.2239940 37470432 PMC10399483

[6] Chai Q Wang L Liu CH Ge B. New insights into the evasion of host innate immunity by *Mycobacterium tuberculosis*. Cell Mol Immunol. (2020) 17:901–13. doi: 10.1038/s41423-020-0502-z, PMID: 32728204 PMC7608469

[7] Mohammadi MJ Farhadi M Ghanbari S Asban P Kiani F Taherian M. Ecological risk assessment of heavy metals in urban dust in Iran: a systematic review and meta-analysis. Toxicol Rep. (2023) 11:471–80. doi: 10.1016/j.toxrep.2023.11.007, PMID: 38075013 PMC10708959

[8] Skalny AV Lima TRR Ke T Zhou JC Bornhorst J Alekseenko SI. Toxic metal exposure as a possible risk factor for COVID-19 and other respiratory infectious diseases. Food Chem Toxicol. (2020) 146:111809. doi: 10.1016/j.fct.2020.111809, PMID: 33069759 PMC7563920

[9] Khalaf EM Taherian M Almalki SG Asban P Kareem AK Alhachami FR. Relationship between exposure to heavy metals on the increased health risk and carcinogenicity of urinary tract (kidney and bladder). Rev Environ Health. (2023). doi: 10.1515/reveh-2022-0245, PMID: 37076952

[10] Juttukonda LJ Berends ETM Zackular JP Moore JL Stier MT Zhang Y. Dietary manganese promotes staphylococcal infection of the heart. Cell Host Microbe. (2017) 22:531–542.e8. doi: 10.1016/j.chom.2017.08.009, PMID: 28943329 PMC5638708

[11] Seth P Husain MM Gupta P Schoneboom A Grieder BF Mani H. Early onset of virus infection and up-regulation of cytokines in mice treated with cadmium and manganese. Biometals. (2003) 16:359–68. doi: 10.1023/a:1020682716212 12572694

[12] Kaur R Rawal R. Influence of heavy metal exposure on gut microbiota: recent advances. J Biochem Mol Toxicol. (2023) 37:e23485. doi: 10.1002/jbt.23485, PMID: 37593904 PMC13546941

[13] Tchounwou PB Ishaque AB Schneider J. Cytotoxicity and transcriptional activation of stress genes in human liver carcinoma cells (HepG2) exposed to cadmium chloride. Mol Cell Biochem. (2001) 222:21–8. doi: 10.1023/A:1017922114201 11678604

[14] Balali-Mood M Naseri K Tahergorabi Z Khazdair MR Sadeghi M. Toxic mechanisms of five heavy metals: mercury, Lead, chromium, cadmium, and arsenic. Front Pharmacol. (2021) 12:12. doi: 10.3389/fphar.2021.643972, PMID: 33927623 PMC8078867

[15] Aschner M Erikson KM Dorman DC. Manganese dosimetry: species differences and implications for neurotoxicity. Crit Rev Toxicol. (2005) 35:1–32. doi: 10.1080/10408440590905920, PMID: 15742901

[16] Qian K Shan L Shang S Li T Wang S Wei M. Manganese enhances macrophage defense against *Mycobacterium tuberculosis* via the STING-TNF signaling pathway. Int Immunopharmacol. (2022) 113:109471. doi: 10.1016/j.intimp.2022.109471, PMID: 36435065

[17] Shan L Wang Z Wu L Qian K Peng G Wei ML. Statistical and network analyses reveal mechanisms for the enhancement of macrophage immunity by manganese in *Mycobacterium tuberculosis* infection. Biochem Biophys Rep. (2024) 37:101602. doi: 10.1016/j.bbrep.2023.101602, PMID: 38155943 PMC10753046

[18] Pelders J Nelson G. Socio-demographic contributors to health and safety of mine workers in South Africa. Work. (2019) 64:67–76. doi: 10.3233/wor-192969, PMID: 31561403

[19] Chen Y Chen Y-q Zhang Q. Association between vitamin D and insulin resistance in adults with latent tuberculosis infection: results from the National Health and nutrition examination survey (NHANES) 2011–2012. J Infect Public Health. (2022) 15:930–5. doi: 10.1016/j.jiph.2022.07.007, PMID: 35878516

[20] Milton B Krewski D Mattison DR Karyakina NA Ramoju S Shilnikova N. Modeling U-shaped dose-response curves for manganese using categorical regression. Neurotoxicology. (2017) 58:217–25. doi: 10.1016/j.neuro.2016.10.001, PMID: 27720796

[21] Kelliher JL Kehl-Fie TE. Competition for manganese at the host-pathogen Interface. Prog Mol Biol Transl Sci. (2016) 142:1–25. doi: 10.1016/bs.pmbts.2016.05.002, PMID: 27571690

[22] Wang C Zhang R Wei X Lv M Jiang Z. Metalloimmunology: the metal ion-controlled immunity. Advances in immunology in China-part B. Adv Immunol. (2020) 145:187–241. doi: 10.1016/bs.ai.2019.11.007, PMID: 32081198

[23] Crossgrove J Zheng W. Manganese toxicity upon overexposure. NMR Biomed. (2004) 17:544–53. doi: 10.1002/nbm.931, PMID: 15617053 PMC3980863

[24] Oh J Shin SH Choi R Kim S Park HD Kim SY. Assessment of 7 trace elements in serum of patients with nontuberculous mycobacterial lung disease. J Trace Elem Med Biol. (2019) 53:84–90. doi: 10.1016/j.jtemb.2019.02.004, PMID: 30910213

[25] Chen X Liu Z Ge X Luo X Huang S Zhou Y. Associations between manganese exposure and multiple immunological parameters in manganese-exposed workers healthy cohort. J Trace Elem Med Biol. (2020) 59:126454. doi: 10.1016/j.jtemb.2020.126454, PMID: 31954213

[26] Nakata A Araki S Park SH Park JT Kim DS Park HC. Decreases in CD8+ T, naive (CD4+CD45RA+) T, and B (CD19+) lymphocytes by exposure to manganese fume. Ind Health. (2006) 44:592–7. doi: 10.2486/indhealth.44.592, PMID: 17085920

[27] Antonini JM Zeidler-Erdely PC Young S-H Roberts JR Erdely A. Systemic immune cell response in rats after pulmonary exposure to manganese-containing particles collected from welding aerosols. J Immunotoxicol. (2012) 9:184–92. doi: 10.3109/1547691x.2011.650733, PMID: 22369286

[28] Liu CH Liu H Ge B. Innate immunity in tuberculosis: host defense vs. pathogen evasion. Cell Mol Immunol. (2017) 14:963–75. doi: 10.1038/cmi.2017.88, PMID: 28890547 PMC5719146

[29] Wang Y Sun Q Zhang Y Li X Liang Q Guo R. Systemic immune dysregulation in severe tuberculosis patients revealed by a single-cell transcriptome atlas. J Infect. (2023) 86:421–38. doi: 10.1016/j.jinf.2023.03.020, PMID: 37003521

[30] Flynn JL Chan J. Immune cell interactions in tuberculosis. Cell. (2022) 185:4682–702. doi: 10.1016/j.cell.2022.10.025 36493751 PMC12162144

[31] Mthembu M Bowman KA Davies LRL Khuzwayo S Mazibuko L Bassett T. Discrepancy between Mtb-specific IFN-gamma and IgG responses in HIV-positive people with low CD4 counts. EBioMedicine. (2023) 90:104504. doi: 10.1016/j.ebiom.2023.104504, PMID: 36870197 PMC9996381

[32] Sonar SA Watanabe M Nikolich J. Disorganization of secondary lymphoid organs and dyscoordination of chemokine secretion as key contributors to immune aging. Semin Immunol. (2023) 70:101835. doi: 10.1016/j.smim.2023.101835, PMID: 37651849 PMC10840697

[33] Azizov V Hubner M Frech M Hofmann J Kubankova M Lapuente D. Alcohol-sourced acetate impairs T cell function by promoting cortactin acetylation. iScience. (2023) 26:107230. doi: 10.1016/j.isci.2023.107230, PMID: 37485352 PMC10362326

[34] Järup L Berglund M Elinder CG. Health effects of cadmium exposure--a review of the literature and a risk estimate. Scand J Work Environ Health. (1998) 24:1–51. 9569444

[35] Hart BA Potts RJ Watkin RD. Cadmium adaptation in the lung – a double-edged sword? Toxicology. (2001) 160:65–70. doi: 10.1016/s0300-483x(00)00436-4 11246125

[36] Mannino DM. Urinary cadmium levels predict lower lung function in current and former smokers: data from the third National Health and nutrition examination survey. Thorax. (2004) 59:194–8. doi: 10.1136/thorax.2003.012054, PMID: 14985551 PMC1746977

[37] Mitra P Sharma S Purohit P Sharma P. Clinical and molecular aspects of lead toxicity: an update. Crit Rev Clin Lab Sci. (2017) 54:506–28. doi: 10.1080/10408363.2017.1408562 29214886

[38] Han B Garcia-Mendoza D van den Berg H van den Brink NW. Modulatory effects of Pb (2+) on virally challenged chicken macrophage (HD-11) and B-lymphocyte (DT40) cell lines in vitro. Environ Toxicol Chem. (2020) 39:1060–70. doi: 10.1002/etc.4702, PMID: 32124477 PMC7277059

[39] Cao J Xu X Zhang Y Zeng Z Hylkema MN Huo X. Increased memory T cell populations in Pb-exposed children from an e-waste-recycling area. Sci Total Environ. (2018) 616-617:988–95. doi: 10.1016/j.scitotenv.2017.10.220, PMID: 29096958

[40] Gardner RM Nyland JF Silbergeld EK. Differential immunotoxic effects of inorganic and organic mercury species in vitro. Toxicol Lett. (2010) 198:182–90. doi: 10.1016/j.toxlet.2010.06.015, PMID: 20600710 PMC4160064

[41] Shao S Zhang Z Feng L Liang L Tong Z. Association of Blood Inflammatory Biomarkers with clinical outcomes in patients with AECOPD: an 8-year retrospective study in Beijing. Int J Chron Obstruct Pulmon Dis. (2023) 18:1783–802. doi: 10.2147/copd.S416869, PMID: 37608836 PMC10441637

[42] Majeed M Nagabhushanam K Prakasan P Mundkur L. Can selenium reduce the susceptibility and severity of SARS-CoV-2?-a comprehensive review. Int J Mol Sci. (2022) 23:4809. doi: 10.3390/ijms23094809, PMID: 35563199 PMC9105991

[43] Akkaş İ Ince N Sungur MA. Serum trace element and heavy metal levels in patients with sepsis. Aging Male. (2020) 23:222–6. doi: 10.1080/13685538.2020.1740200 32183594

